# Effect of superior laryngeal nerve block in alleviating sore throat after application of i-gel supraglottic airway: a randomized controlled trial

**DOI:** 10.1186/s12871-023-02287-7

**Published:** 2023-10-05

**Authors:** Zhu Lv, Xinyi Huang, Jinxia Cai, Zijun Zhou, Linglin Gao, Weijian Wang, Jiehao Sun, Yizhao Pan

**Affiliations:** Department of Anesthesiology, 1st affiliated hospital, Wenzhou Medical University, Wenzhou, Zhejiang China

**Keywords:** Superior laryngeal nerve block, Postoperative sore throat, Supraglottic airway device, i-gel

## Abstract

**Background:**

Postoperative sore throat (POST) is a common complaint after supraglottic airway device (SAD) application. Internal branch of the superior laryngeal nerve (iSLN) block has the potential to alleviate POST. The aim of this trial was to explore the effect of iSLN block in alleviating sore throat, as well as to identify the potential risk factors for POST after SAD insertion.

**Methods:**

One hundred thirty-four patients scheduled for elective gynecological surgery were randomized to either group T: tetracaine syrup (1%) for local lubrication on i-gel supraglottic device (*n* = 67) or group B: i-gel insertion with water based lubricant on it and followed by bilateral iSLN block (ropivacaine, 0.375%, 2 ml for each side) (*n* = 67). Under ultrasound guidance, iSLN was exposed below thyrohyoid membrane. The primary outcome was the intensity of sore throat at 6 h after surgery. In addition, POST score at 0.5 h and 24 h, the severity of postoperative swallowing discomfort, acoustic analysis and complications were measured.

**Results:**

Compared with tetracaine syrup for local lubrication, iSLN block resulted in a reduced intensity of POST at 0.5 h (*P* = 0.044, OR = 1.99, 95%CI 1.02 to 3.88) and 6 h (*P* < 0.001, OR = 5.07, 95%CI 2.53 to 10.14) after surgery, as well as less severity of swallowing discomfort (*P* < 0.001, OR = 2.21, 95%CI 1.63 to 2.99) and cough (*P* = 0.039, OR = 1.97, 95%CI 1.04 to 3.73). The patients after iSLN block presented lower jitter and shimmer value in acoustic analysis at 6 h after surgery (*P* < 0.001).

**Conclusions:**

iSLN block was effective in alleviating POST, improving voice function, as well as reducing postoperative swallowing discomfort and coughing.

**Trial registration:**

Chinese Clinical Trial Registry (ChiCTR2000037974) on 8th Sept 2020.

**Supplementary Information:**

The online version contains supplementary material available at 10.1186/s12871-023-02287-7.

## Background

I-gel is a supraglottic airway device (SAD) widely used for airway management during general anesthesia. Meanwhile, postoperative sore throat (POST) is one of the frequent complaints among patients using SAD. Although most of POST is typically self-limiting, it is rated as the eighth most undesirable outcome in the postoperative period [1]. Recent reports found that the incidence of POST caused by i-gel supraglottic airway was approximately 49% [2]. Pharyngeal soft tissue abrasions cause delayed feed and prolonged hospitalization [3]. Severe POST is even associated with postoperative insomnia, memory loss [4, 5]. Anesthetic agent spray or gargle, steroids administration, are proposed to reduce the incidence of sore throat, but yield controversy on the efficacy of the treatments [2, 6, 7]. Except for local pharyngeal mucous injury, other risk factors such as age, surgical duration, gender were also involved in initiating POST after SAD insertion [3, 8, 9].

Internal branch of the superior laryngeal nerve (iSLN) predominantly supplies supraglottis and pharyngeal mucosa. There is increasing recognition that the iSLN is superficially positioned and therefore easily blocked in daily clinical practice. Prior research revealed that iSLN block could effectively alleviate POST after endotracheal tubes (ETT) [10–12]. Although POST caused by ETT intubation is different with that by SAD insertion, some causes from them are overlapped. Both are the result of direct trauma by rigid materials inserted into the upper airway, and pharyngeal and laryngeal soft tissue abrasions [13, 14].

This prospective randomized clinical trial was performed to investigate the efficacy and safety of iSLN block in relieving POST after SAD insertion in elective gynecologic surgery. Furthermore, we also attempted to explore and identify the risk factors for POST, to provide a better understanding of prediction, in order to allow individualized management after supraglottic airway device insertion in the future.

## Methods

### Design and study setting

This study was a prospective randomized clinical trial. The cases were recruited at the 1st Affiliated Hospital of Wenzhou Medical University from 15th Sept 2020 to 28th Feb 2022. The Ethics Committee of the First Affiliated Hospital, Wenzhou Medical University granted ethical approval (No.2020–178) and the trial was prospectively registered in the Chinese Clinical Trial Registry on 8th Sept, 2020 (ChiCTR2000037974). This study was conducted in adherence to the principles of the Helsinki Declaration and complied with CONSORT standards. Informed consent in this study was acquired from patients before participation.

### Participants

Patients were admitted if they complied with all the criteria listed below: ① adult female ≥ 20 years of age ② ASA grade I- II ③ general anesthesia after supraglottic airway device insertion ④ non-malignant laparoscopic gynecological surgery. Exclusion criteria were emergency surgery; massive intraoperative hemorrhage; preoperative hoarseness or sore throat; renal, hepatic, neurological, or cardiovascular dysfunction before surgery.

### Randomization and allocation

A random number table was created by Excel. The sequence was kept in sealed opaque envelopes by Gao who was not engaged in patient assessment. The patients in the Group B received bilateral iSLN block after i-gel supraglottic airway insertion. The patients in the Group T received tetracaine syrup (1%) prior to i-gel insertion.

### Interventions

After the patient's admission to the operating room, routine monitoring of blood pressure, heart rate (HR), electrocardiogram (ECG), and pulse oxygen saturation (SpO_2_) was performed. Prior to anesthetic induction, doctor Zhou who was not included in the patient assessment collected voice recordings for the included patients. Anesthesia was induced by sufentanil 0.4 mg kg^−1^, propofol 2.0 mg kg^−1^, and rocuronium 0.06 mg kg^−1^. After loss of eyelash reflex, the attending doctor left the operative room. Doc Sun who was unaware of the assessment opened the sealed envelope. According to grouping, the patients in the Group B received bilateral iSLN block after i-gel supraglottic airway insertion. The i-gel was lubricated with the water based lubricant (KL-250 jelly, Keppler Medical Appliances Co. Ltd, Hangzhou, China). The patients in the Group T received tetracaine syrup (1%) on i-gel supraglottic device for lubrication. The SAD was inserted 3 min after completion of induction.

I-gel insertion approach: the manufacturer's weight-based recommendations were followed for size selection. The size selection for i-gel was also considered in conjunction with clinical assessment of the patient’s anatomy. The patient was in the sniffing position with head extended and neck flexed. The doctor introduce the leading soft tip into the mouth with the direction towards the hard palate. Glide the device along the hard palate until a definitive resistance was felt. The insertion procedure was performed gently with slight adjustment in the mouth. The insertion was attempted once in all patients.

After i-gel insertion, the neck was covered by gauze to blind the evaluators and patients. The covered gauze was not removed until the ending of the evaluation at 24 h after the surgery. After that, the attending anesthesia doctor who was in charge of general anesthesia was called to return to the operative room. Mechanical ventilation was performed with a controlled tidal volume of 6-8 ml/kg and respiratory rate of 12 per minute. Propofol, remifentanil, and sevoflurane inhalation (constant at MAC 0.7) were used to maintain anesthesia. Meanwhile bispectral index (BIS) monitoring was used for guiding anesthetic administration. Flurbiprofen axetil (50 mg) was given as postoperative analgesia.

Voice recordings were obtained prior to anesthetic induction (0 h), 6 h and 24 h after surgery. Patients were inquired about the severity of the POST at 0.5 h, 6 h and 24 h after surgery. The intensity of POST was assessed with a quadrant scaling as follows: 0, no sore throat; 1, sore throat without complaint before inquiry; 2, sore throat with complaint; 3, severe sore throat and refusal to swallow. Sore throat was defined when POST grading > 1.

Agitation was defined as the case who can not calm down even after verbal reminder. The intensity of cough was graded from 0 to 2. (0: none, 1: slight cough, 2: severe cough with body movement). The severity of cough and the incidence of agitation were recorded during the emergence time. The intensity of swallowing discomfort was recorded at 6 h after operations (NRS score: 0–10, 0: no pain, 10: the most severe pain and refusal to swallow). Anesthesiologist, patients, outcome assessors were blinded to the group allocation.

### iSLN block under ultrasound exposure

The patient was positioned with the neck extended. An oblique parasagittal scan was performed between the levels of the hyoid and thyroid cartilage. A 6.0—12.0 MHz linear ultrasound probe (GE Logiq e) was placed with compression to identify the thyrohyoid membrane. A 26G needle (TWLB, Kangdelai Medical Appliances Co. China) was inserted via the out-of-plane technique under ultrasound guidance until the needle tip entered the target space, which was close to superior laryngeal artery below thyrohyoid membrane. 4 ml ropivacaine solution (0.375%, 2 ml each side) was used to block bilateral internal branch of the superior laryngeal nerve..

### Acoustic analysis

After a deep breath, a sustained vowel /i:/ was pronounced. The voice was recorded for analysis after collection with a microphone (Yamaha UR22C-R-PACK, Japan) at a 45° angle positioned 5 cm from the patient's mouth. The voice records were analyzed using the phonetic application (PRAAT v6.2.19. Phonetic Science, University of Amsterdam, Netherland), which measured jitter and shimmer for each patient. Jitter refers to the short-term variations in the fundamental frequency, and shimmer represents the short-term variations in the amplitude of sound waves between contiguous glottal cycles.

### Outcomes

Demographic characteristics were collected, including age, BMI, neck circumference, thyromental distance and interincisor distance.

The primary outcome measure was the sore throat intensity at 6 h. The secondary outcome measures included: ① the POST intensity at 0.5 h and 24 h, ② swallowing discomfort, ③ acoustic analysis including jitter and shimmer, ④ the episodes of agitation, cough.

### Statistical analysis

Continuous data were assessed for skewness by using the Shapiro–Wilk test and were expressed as mean ± standard deviation (SD) or median (IQR). Categorical variables were presented as the number (%). Numerical variables were analyzed using independent samples t-test or Mann–Whitney U test. Categorical variables were compared using Pearson χ^2^ test or Fisher’s exact test. Generalized linear models were used to compare the intensity of POST between the group B and group T.

Univariate analysis using logistic regression was performed to calculate the predictors of POST as odds ratios (ORs) with 95% confidence intervals (CIs). If these variables yielded *P* < 0.1, they were incorporated into the subsequent multivariate adjusted ordinal logistic regression analysis. *P* < 0.05 was considered as statistically significant. IBM SPSS Statistics (version. 23.0, IBM Corp, Armonk, NY. USA) and R package (version: 4.3.1) was used for the statistical analysis.

In our preliminary investigation based on 17 cases per group, it was showed that the mean score of POST evaluated by the quadrant scaling was 1.12 ± 0.82 at 6 h following i-gel insertion (grade 0–3: 4/8/4/1). Correspondingly, the quadrant scale of the cases with nerve block was grade 0–3: 6/10/1/0. We calculated the sample size based on the Wilcoxon-Mann–Whitney test. For an error of 0.05 and 80% power, a sample size of 61 patients per group would be required. In order to compensate for dropouts, 140 patients were enrolled in this trial.

## Results

**Fig. 1:**
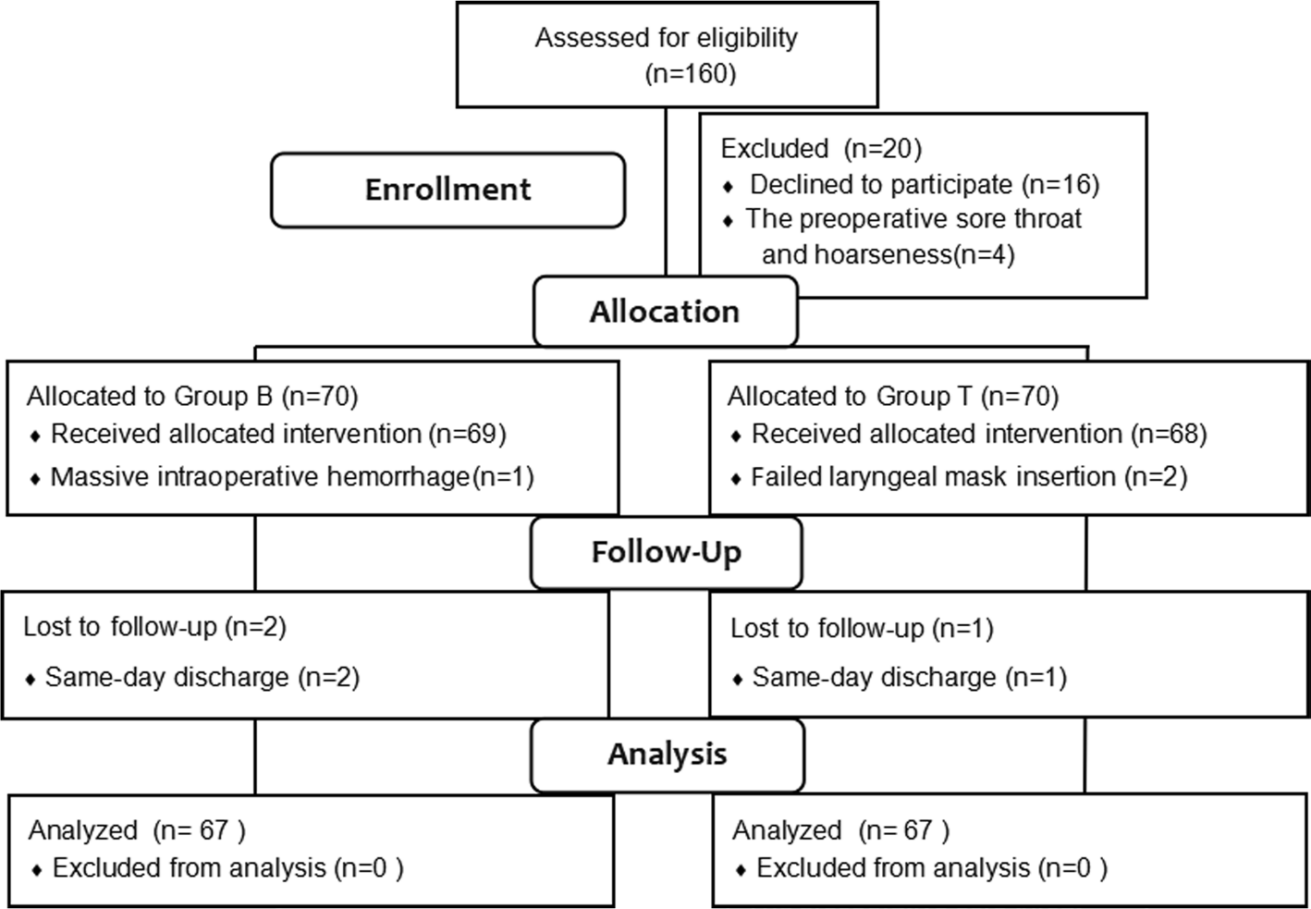
Consolidated Standards of Reporting Trials flow diagram. Group T: tetracaine syrup for local lubrication; Group B: bilateral iSLN block

A total of 160 subjects were screened for eligibility at the 1st Affiliated Hospital of Wenzhou Medical University. 26 cases were excluded from the study. The CONSORT flow of participants through the study is visualized in Fig. 1. Demographic characteristics are summarized in Table 1.

**Table 1:** Baseline and perioperative characteristics

Variables	Group T (*n* = 67)	Group B (*n* = 67)
Age (years)	54.0 (46.0, 59.0)	55.0 (49.0, 58.0)
BMI (kg·m^−2^)	23.8 (22.5, 24.8)	23.6 (22.4, 24.6)
ASA classification(I/II)	55/12	56/11
Duration of surgery (min)	91.0 (58.0, 115.0)	87.0 (56.0, 116.0)
Duration of anesthesia(min)	99.0 (71.0, 122.0)	98.0 (64.0, 125.0)
Mallampati grade(I/II/III)	33/28/6	33/22/12
Circle neck(cm)	35.0 (32.5, 37.4)	34.6 (32.0, 36.8)
Thyromental distance(cm)	68.0 (65.0, 72.0)	68.0 (65.0, 72.0)
Interincisor distance(cm)	69.0 (66.0, 72.0)	70.0 (65.0, 71.0)
Pharyngeal suctioning times (1/2/3)	38/22/7	34/27/6
Blood sputum	6 (9.0%)	4 (6.0%)

Group T: tetracaine syrup for local lubrication; Group B: bilateral iSLN block. *Abbreviations*: *ASA* American society of anesthesiologist, *BMI* body mass index

### The intensity of POST

The intensity of POST is shown in Table 2. The iSLN block presented less intensity in POST compared with the group T at 6 h (*P* < 0.001) and 0.5 h (*P* = 0.044) after surgery.

**Table 2:** Outcomes and side effect of the two test groups

Variables	Group T (*n* = 67)	Group B (*n* = 67)	*P* value	OR (95%CI)
The intensity of POST grade: 0:1:2:3				
0.5 h	29:29:9:0	37:30:0:0	0.044	1.99 (1.02–3.88)
6 h	14:19:29:5	24:39:4:0	< 0.001	5.07 (2.53–10.14)
24 h	57:8:2:0	63:4:0:0	0.095	2.81 (0.84–9.48)
Secondary outcomes:				
Cough intensity: 0:1:2	23:28:16	29:34:4	0.039	1.97 (1.04–3.73)
PONV n (%)	24 (35.8%)	25 (37.3%)	0.850	1.07 (0.53–2.16)
Agitation n (%)	38 (56.7%)	30 (44.8%)	0.167	0.62 (0.31–1.22)
Swallowing discomfort	4 (3, 5)	3 (3, 4)	< 0.001	2.21 (1.63–2.99)

Group T: tetracaine syrup for local lubrication; Group B: bilateral iSLN block

*Abbreviations*: *OR* odds ratios, *CI* confidence interval, *POST* Postoperative sore throat, *PONV* Postoperative nausea and vomiting

Group B led to lower NRS score in swallowing discomfort (*P* < 0.001) and less intensity in cough (*P* = 0.039) (Table 2). There was no difference in agitation or PONV (Table 2).

### Risk factors for POST

**Fig. 2:**
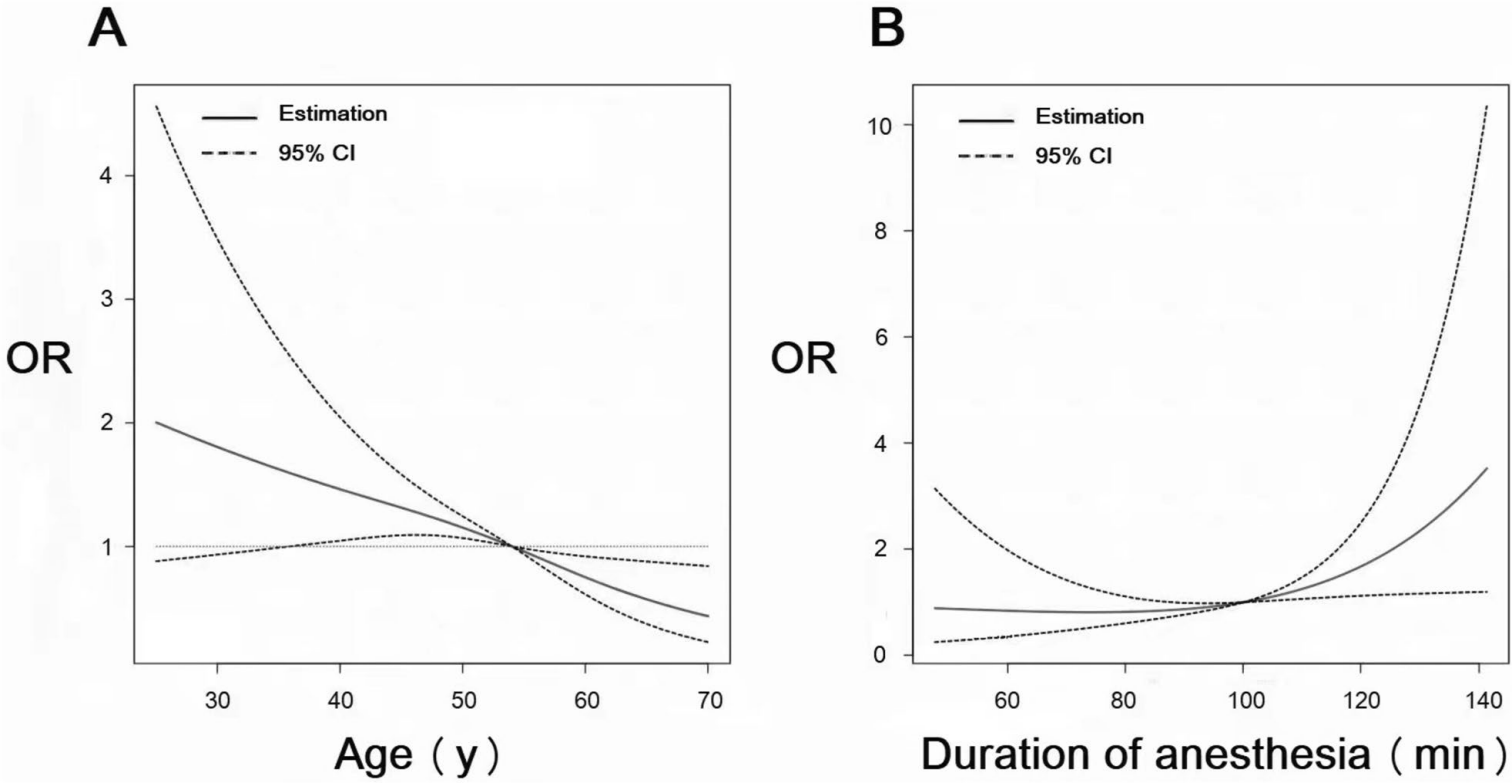
Estimated associations between POST and age, duration of anesthesia. POST: Postoperative sore throat

Univariate logistic regression analyses identified two other independent risk factors for POST: longer duration of anesthesia and younger age (Table 3). Estimated associations between POST and age, duration of anesthesia were shown from non-linear spline models (Fig. 2 A-B).

**Table 3:** Univariate logistic regression analysis of risk factors for POST

Variables	OR (95% Cl)	*P* value
Age	0.97 (0.93–1.01)	0.093
Duration of anesthesia	1.02 (1.00–1.03)	0.015
Blood sputum	1.76 (0.47–6.64)	0.401
Circle neck circumference	0.93 (0.82–1.06)	0.273
Thyromental distance	0.95 (0.86–1.05)	0.339
Interincisor distance	0.98 (0.89–1.08)	0.689
BMI	0.85 (0.64–1.11)	0.233
ASA classification	0.66 (0.22–1.92)	0.441

*Abbreviations*: *OR* odds ratios, *CI* confdence interval, *POST* Postoperative sore throat, *ASA* American society of anesthesiologist, *BMI* body mass index

After adjustment of multivariate ordinal logistic analysis, the association between duration of anesthesia and POST was still robust (*P* = 0.006, OR = 1.02, 95%CI 1.01 to 1.04).

### Change of voice function

**Fig. 3:**
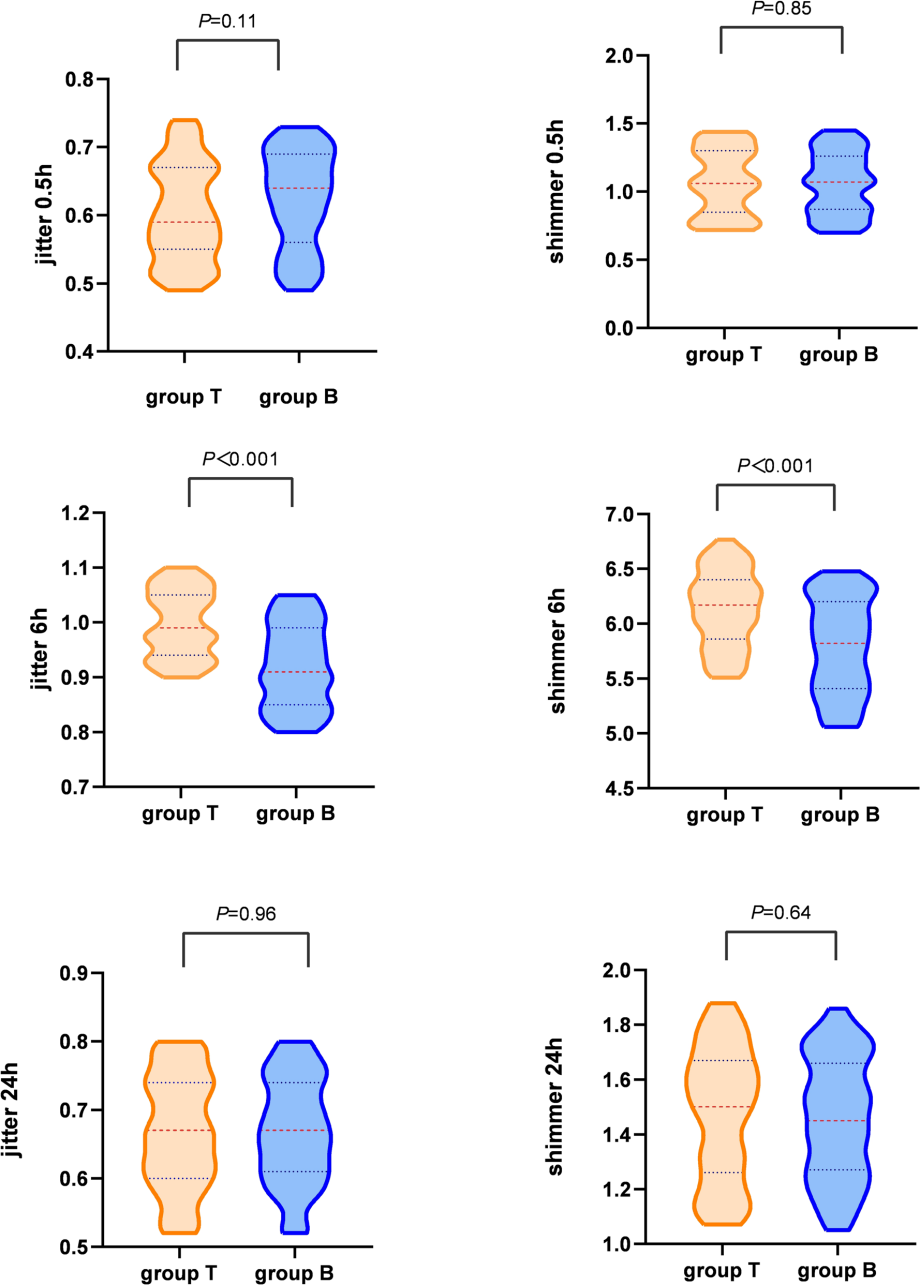
Variation of voice function including jitter and shimmer in both T and B groups. Group T: tetracaine syrup for local lubrication; Group B: bilateral iSLN block

In comparison with the group T, group B provided better voice function, which was characterized in less elevation in the jitter (*P* < 0.001) and shimmer (*P* < 0.001) at 6 h (Fig. 3).

## Discussion

In this trial, we demonstrated that the iSLN block was more effective in lowering the severity of POST, reducing swallowing discomfort and subsequent cough after elective gynecological surgery. Moreover, bilateral iSLN block also yielded a better effect on voice function.

Multiple interventions were used to decrease POST in previous research, such as NSAIDs administration, local anesthetics lubrication [15, 16]. However, the analgesic effects of the local anesthetics lubrication were far below expectations [17, 18]. Despite of NSAIDs use, the incidence of POST could be still up to 20% [15].

POST caused by SAD could be the result of direct trauma by rigid materials inserted into the upper airway, physical tensile stresses imposed by the cushions or gel [14, 15]. SAD provoked irritation on supraglottic areas, causing ischemic lesion and pharyngeal and laryngeal soft tissue abrasions.

The iSLN provides sensory innervation of the mucosa surrounding the pharynx above glottis fissure [19]. It has been well noted that the iSLN is well exposed in ultrasound images and easily be blocked, even in the pathological obesity [20].

The iSLN is considered as a pure sensory nerve to predominantly supply the pharyngeal structure close to vocal cord [21]. Our prior trial concluded that the iSLN block could effectively relieve POST without subsequent dysphonia [12]. In addition, the iSLN block can minimize the hemodynamic change caused by airway stimulation.

Postoperative dysphonia has been reported after SAD insertion [22, 23], though the phonation alteration was temporary. The balance of supraglottic structure is crucial in the pronunciation. The iSLN has no motor effect on vocal muscle [24]. The vibration of the vocal cord had no change after iSLN block. Due to POST, the cases without iSLN block dare not to pronounce when follow-up. So the variations of intensity and frequency of the voice was low, which was characterized as lower jitter and shimmer.

Duration of anesthesia was correlated with POST. Studies have shown that mechanical ventilation for more than 1.5 h can cause submucosal congestion and damage in the larynx [25]. It is a debate whether age is a risk factor [3]. Surgery in elders is prone to more complex, so elderly is more likely to experience POST. While in other reports, more POST was found in younger [26, 27]. In our trial, it was failed to conclude the correlation between POST and age after adjustment of multivariate analysis.

Difficult airway has the potential to modify the occurrence of POST. Neck circumference, thyromental distance and interincisor distance are used to predict the probability of difficult airway. A higher thyromental distance, to our common knowledge, provides a better condition for SAD insertion, and hence reduces the probability of POST. The thyromental distance of the included patients in the trial was 62-80 mm, whereas thyromental distance less than 60 mm was generally considered to be a risk factor for difficult intubation. Thus it failed to gain the correlation between neck circumference, thyromental distance, interincisor distance and POST in the trial. In addition, the presence of bloody sputum during extubation is resulted from pharyngeal trauma. Nonetheless, our study failed to detect the association with POST.

### Limitation

POST is a subjective experience and repeatedly assessed in the trial. The questioning of a patient about the intensity of POST may initiate recall bias. Gender was a risk factor for POST in the previous literature [3, 8, 9]. However, all of the patients included in the trial were female.

## Conclusion

The application of iSLN block was effective in reducing the intensity of POST, improving the quality of voice function.

### Supplementary Information

**Additional file 1.**

## Data Availability

All data generated or analyzed during this study are included in this published article [and its supplementary information files].

## Abbreviations

POST: Postoperative sore throat
SAD: Supraglottic airway device
iSLN: Internal branch of the superior laryngeal nerve
NSIADs: Nonsteroidal anti-inflammatory drugs
PONV: Postoperative nausea and vomiting
NRS: Numerical rating scale
BIS: Bispectral index
BMI: Body mass index
ETT: Endotracheal tubes
OR: Odds ratio
CI: Confidence interval
